# Emotional eating as predictor of weight loss 2 years after Roux‐en‐Y gastric bypass

**DOI:** 10.1111/cob.12458

**Published:** 2021-05-30

**Authors:** Marleen M. Romeijn, Jessica Schellekens, Daniëlle S. Bonouvrie, Loes Janssen, François M. H. van Dielen, Wouter K. G. Leclercq, Marieke van de Wal

**Affiliations:** ^1^ Department of Surgery Máxima Medical Centre Veldhoven Netherlands; ^2^ Research School NUTRIM, Department of Surgery Maastricht University Medical Centre Maastricht Netherlands; ^3^ Faculty of Social and Behavioural Sciences Tilburg University Netherlands; ^4^ Department of Medical Psychology Máxima Medical Centre Veldhoven Netherlands

**Keywords:** bariatric surgery, emotional eating, Roux‐en‐Y gastric bypass, weight loss

## Abstract

There has been little agreement on the predictive value of emotional eating on weight loss outcomes after bariatric surgery. The aim of this study was to examine the predictive value of preoperative emotional eating, in response to clearly labelled emotions and diffuse emotions, on excess weight loss (EWL) and total weight loss (TWL) 2 years after Roux‐en‐Y gastric bypass (RYGB). All participants included in this retrospective cohort study were screened for RYGB surgery by a multidisciplinary team. The level of emotional eating was derived from the Dutch Eating Behaviour Questionnaire (DEBQ); the level of psychological variables from the Symptom Checklist‐90. Participants were clustered, based on their DEBQ score, in high and low emotional eaters. Multiple linear regression analyses were performed to examine the association between preoperative emotional eating and EWL, and TWL. There were no significant differences in EWL of the 172 included participants, defined as either high or low emotional eaters (EWL 82.7% ±18.2 versus 82.4% ±21.3, respectively). Based on the regression analysis, emotional eating was not significantly associated with EWL, nor with TWL. When corrected for psychological, demographic and biological variables, preoperative emotional eating in response to diffuse emotions negatively affected EWL (*β* = −0.16, *P* = 0.048), although this was not applicable for TWL. Preoperative emotional eating does not seem to influence EWL, nor TWL 2 years after RYGB. Since this study faced multiple limitations, further investigation is required regarding the predictive value of emotional eating.

**Abbreviations**
BMI: body mass index
DEBQ: Dutch eating behaviour Questionnaire
EWL: excess weight loss
RYGB: Roux‐en‐Y‐Gastric Bypass
SCL‐90: symptom checklist‐90
TWL: total weight loss

**What is already known about this subject**

- Emotional eating can occur in response to clearly labelled and diffuse emotions
- Emotional eating is frequently observed in patients undergoing bariatric surgery

**What this study adds**

- This study describes the impact of preoperative emotional eating on weight loss after bariatric surgery
- This study shows that emotional eating does not affect excess weight loss, nor total weight loss after 2 years

## 1 INTRODUCTION

Despite the impressive effects of bariatric surgery on weight loss and obesity related comorbidities, 25% to 35% of patients do not respond well to this intervention.^1^, ^2^ These patients may experience insufficient weight loss or regain a substantial amount of weight after initial adequate weight loss.^3^ Insufficient weight loss is expressed as a primary non‐response and is often defined as <50% excess weight loss (EWL) up to 2 years after bariatric surgery.^4^ Given the high prevalence of the above, predictors of non‐response after bariatric surgery have been an area of great interest.

Emotional eating is defined as a maladaptive coping strategy where emotional arousal leads to an excessive food intake.^5^ An excessive food intake would hypothetically counteract postoperative weight loss and thereby induce a non‐response. Emotional eating is reported in 38% to 59% of bariatric candidates and occurs in response to clearly labelled emotions (eg, anger and fear) and diffuse emotions (eg, boredom and restlessness).^6^, ^7^ Compared to clearly labelled emotions, diffuse emotions are often more ambiguous, yet both types of emotional eating can be difficult for a patient to identify.^5^

When reviewing literature, there are contradictory findings about the impact of emotional eating on weight loss outcomes.^8^, ^9^, ^10^, ^11^, ^12^ Monpellier showed that a postoperative change in emotional eating was negatively related to the percentage of total weight loss (TWL) up to 4 years after Roux‐en‐Y gastric bypass (RYGB), but preoperative emotional eating did not predict a non‐response.^8^ On the contrary, Miller‐Matero showed that preoperative emotional eating was associated with less TWL 1 year after surgery.^9^ Similarly, Castellini showed that higher levels of preoperative emotional eating predicted lower excess body mass index (BMI) weight loss 1 year after surgery.^10^

The primary aim of the current study was to investigate the predictive value of preoperative emotional eating on EWL and TWL 2 years after RYGB. The secondary aim of this study was to explore the differential impact of emotional eating in response to clearly labelled versus diffuse emotions on EWL and TWL. Based on the studies that found a negative association between emotional eating and postoperative weight loss, it was hypothesized that the level of preoperative emotional eating was negatively associated with EWL and TWL.^9^, ^10^, ^12^

## 2 MATERIALS AND METHODS

### 2.1 Study cohort

Data from participants that underwent a primary RYGB within our hospital between the 1st of January 2015 and the 31st of December 2015 were analysed in retrospect. All participants were screened for surgery by a multidisciplinary team. To qualify for bariatric surgery, criteria of the 'International Federation for the Surgery of Obesity and Metabolic Disorders' were applied.^13^ Participants were included if they completed the required psychosocial assessments preoperatively, and if they had been to the follow‐up visits 2 years after RYGB. Participants who received psychological interventions pre‐ or postoperatively were not excluded from the study. Pregnancy during the follow‐up period or missing anthropometric data 2 years after surgery were exclusion criteria. All procedures performed within this study were in accordance with the ethical standards of the institutional and/or national research committee and with the 1964 Helsinki declaration and its later amendments or comparable ethical standards. Study procedure was approved by a local Medical Ethics Review Committee (N17.145, date of approval October 6, 2017).

### 2.2 Data collection

Sociodemographic and psychological information concerning gender, age, level of education, work status, marital status, medication use and/or mental healthcare treatment in the past were obtained by use of preoperative screening questionnaires, as well as additional information related to pre‐or postoperative psychological interventions. The aim of these interventions were to implement small behavioural adjustments related to eating behaviour, diet and/or physical activity. The level of emotional eating was assessed prior to the start of a psychological intervention. Information about BMI and weight was obtained by using electronic patient files.

### 2.3 The Dutch Eating Behaviour Questionnaire

The Dutch Eating Behaviour Questionnaire (DEBQ) was used for assessment of emotional eating. The DEBQ is a validated 33‐item self‐report questionnaire that differentiates between emotional eating in response to clearly labelled and diffuse emotions, external eating and restrained eating.^7^ Thirty‐three statements are rated on a 5‐point scale, with responses ranging from 1 (never) to 5 (very often). Total scores for the emotional eating scale range between 13 to 65, whereas for the external and restrained eating scale they range between 10 to 50. Higher scores indicate an eating behaviour which is more pathological. The Dutch version of the DEBQ is of high quality in terms of reliability and validity (*α* = 0.95‐ 0.96).^14^ Participants were clustered, based on their DEBQ score, in high and low emotional eaters. This classification was performed for the categories 'overall emotional eating', 'emotional eating in response to clearly labelled emotions' and 'emotional eating in response to diffuse emotions'. In order to make this classification, gender specific cut‐off scores were applied based on normative data from a Dutch obese population.^7^, ^15^, ^16^ These cut‐off scores can be found in Table S1.

### 2.4 Symptom Checklist‐90

The Symptom Checklist‐90 (SCL‐90) was used for assessment of psychopathology including anxiety and depression. The SCL‐90 is a self‐report questionnaire that measures physical and psychological complaints.^17^ The questionnaire contains eight subscales: agoraphobia, anxiety, depression, somatization, insufficient thinking or acting, distrust and interpersonal sensitivity, hostility and sleep problems. Ninety statements are rated on a 5‐point scale with responses ranging from 1 (not at all) to 5 (extremely). Total SCL‐90 scores are calculated as the sum of the subscale scores and range between 90 to 450. The subscale score for depression ranges between 16 and 80, while for anxiety this ranges between 10 and 50. The Dutch version of the SCL‐90 is of moderate quality in terms of reliability and validity (*α* = 0.80).^18^

### 2.5 Weight change

Weight loss was described as %EWL and was calculated as follows: (initial weight − final weight) / (initial weight − ideal body weight) × 100%. Initial weight was defined as the weight at the moment of preoperative screening. Ideal body weight was based on a BMI of 25 kg/m^2^. Additionally, weight loss was expressed in %TWL and was calculated as follows: ([initial weight ‐ final weight] / initial weight) × 100%. Participants were clustered, based on their %EWL, as primary responders and primary non‐responders. An EWL of ≥50% 2 years after RYGB was considered as a primary response, while an EWL of <50% after 2 years was considered as a primary non‐response.^4^

### 2.6 Statistical analysis

Descriptive statistics were computed for sociodemographic and psychological characteristics. For each type of emotional eating, the associations between the level of emotional eating and covariates (ie, gender, age, initial BMI, marital status, preoperative psychological intervention and the level of preoperative anxiety and depression) were analysed using (non‐parametric) correlations. The internal consistency of the DEBQ and SCL‐90 was assessed by measuring Cronbach's alpha. Independent sample *t*‐tests were performed to examine differences between primary responders and primary non‐responders in preoperative demographic and biological data. An independent sample t‐test was performed to detect differences in %EWL in participants with either high or low scores of emotional eating.

A three‐stage hierarchical multiple linear regression model was applied three times to examine the association between emotional eating (continuous, independent variable) and EWL (continuous, dependent variable), as well as to test whether these associations were independent of other predictors of EWL. In stage one, the primary predictor was entered which was the total score on emotional eating in the first model, the score on emotional eating in response to clearly labelled emotions in the second model, and the score on emotional eating in response to diffuse emotions in the third model. In stage two, psychological covariates (preoperative anxiety and depression) were added. In stage three, demographic and biological covariates (gender, age, initial BMI, marital status, type 2 diabetes mellitus and preoperative psychological intervention) were added. For each model, the 95% confidence interval was calculated and the significance level was set at 5% (*P* <  0.05). The multiple linear regression model was repeated with TWL as dependent variable. All analyses were performed using the program Statistical Package for Social Sciences version number 22.0 (IBM SPSS 22.0).

## 3 RESULTS

The sample set consisted of 302 participants. Two participants were excluded due to pregnancy during 2 year follow‐up. An additional 128 participants were excluded due to missing data during 2 year follow‐up, or due to an incomplete questionnaire that was required during preoperative screening (eg, DEBQ). As a result, 172 participants were included in this study.

The sociodemographic characteristics of the cohort are shown in Table 1. Excluded participants did not differ significantly from the included patients in baseline characteristics for example gender, age and preoperative BMI (data not shown). The mean scores of emotional eating did not differ between the group of responders (32.3 ± 11.8) and non‐responders (30.6 ± 10.1). Non‐responders had a higher BMI (*P* = 0.04) and a higher use of mental healthcare in the past (*P* = 0.02) in comparison to responders. Between high and low emotional eaters, there were no significant differences in EWL with average overall scores of 82.7% ±18.2 and 82.4% ±21.3, respectively. The average score of the DEBQ within each category is illustrated in Table 2.

**TABLE 1:** Characteristics of the study population

	Total n = 172	Primary response^a^ n = 161	Primary non‐response^b^ n = 11	*P* value^f^
Gender, no. of females (%)	144 (83.7)	135 (83.9)	9 (81.8)	.86
Age, years mean ± SD	44.9 ± 10.2	44.6 ± 10.2	50.1 ± 9.7	.09
Initial weight (kg), mean ± SD	120.7 ± 19.3	120.2 ± 19.1	127.9 ± 22.7	.21
Initial BMI (kg/m^2^), mean ±SD	42.4 ± 5.0	42.2 ± 4.8	45.5 ± 7.1	.04^g^
EWL (%), mean ±SD	82.4 ± 20.6	85.5 ± 18.5	44.0 ± 3.7	<.001^g^
TWL (%), mean ±SD	32.7 ± 8.0	33.6 ± 7.4	19.2 ± 4.2	<.001^g^
Change in BMI (kg/m^2^), mean ±SD	13.9 ± 4.4	14.3 ± 4.2	8.9 ± 3.1	<.001^g^
Marital status, no. of married (%)	141 (82)	132 (81.9)	9 (81.8)	.99
Educational level^c^ (%)				
< 6 years	14 (8.2)	14 (8.2)	0 (0)	.31
6‐12 years	133 (77.3)	123 (71.5)	10 (91)	.27
More than 12 years	25 (14.5)	24 (14.9)	1 (9)	.60
Work status (%)				
Employed	112 (65.1)	107 (66.5)	5 (45.5)	.11
Unemployed	60 (34.9)	54 (33.5)	6 (54.5)	.11
Use of mental healthcare (%)	83 (48.3)	74 (46)	9 (81.8)	.02^g^
Preoperative psychological intervention (%)	62 (36)	59 (36.6)	3 (27.3)	.53
Preoperative use of antidepressants (%)	21 (12.2)	19 (11.8)	2 (18.2)	.53
Emotional eating^d^, mean ±SD	32.2 ± 11.7	32.3 ± 11.8	30.6 ± 10.1	.63
Clearly labelled, mean ±SD	21.0 ± 8.4	21.2 ± 4.5	19.0 ± 6.8	.41
Diffuse, mean ±SD	11.2 ± 4.1	11.2 ± 4.1	11.3 ± 3.8	.95
External eating^d^, mean ±SD	28.5 ± 5.7	28.6 ± 5.9	27. 5 ± 2.9	.54
Restrained eating^d^, mean ±SD	31.9 ± 6.5	32.1 ± 6.6	28.8 ± 5.1	.11
Psychoneuroticism^e^, mean ±SD	146.4 ± 39.7	145.9 ± 39.4	154.5 ± 44.3	.49
Depression, mean ±SD	30.9 ± 29.4	30.9 ± 30.3	30.5 ± 11.0	.96
Anxiety, mean ±SD	13.9 ± 4.6	13.9 ± 4.6	14.6 ± 3.9	.66

Abbreviations: BMI, body mass index; EWL, excess weight loss; TWL; total weight loss; SD, standard deviation; no, number.

^a^ Patients with ≥50% EWL 2 years after surgery.

^b^ Patients with <50% EWL 2 years after surgery.

^c^ Six years of education (primary school). Six to 12 years of education (LTS, MAVO, [M]ULO, HAVO, VWO). More than 12 years of education (HBO, WO, post‐HBO/master).

^d^ Measured with the Dutch Eating Behaviour Questionnaire (DEBQ), Cronbach's alpha of 0.75.

^e^ Measured with the Symptom Checklist‐90 (SCL‐90), Cronbach's alpha of 0.68.

^f^ Based on independent samples *t*‐test.

^g^ *P value* ≤ .05.

**TABLE 2:** Mean percentage of EWL and TWL in participants with high and low scores of emotional eating

	High score^a^			Low score^b^					
	DEBQ score, mean ±SD	EWL (%), mean ±SD	TWL (%), mean ±SD	DEBQ score, mean ±SD	EWL (%), mean ±SD	TWL (%), mean ±SD	*P* value^c^	95% CI^c^	Effect size (Cohen's *d*)^c^
Emotional eating overall	3.6 ± 0.5	82.7 ± 18.2	31.8 ± 6.8	2.1 ± 0.7	82.4 ± 21.3	32.9 ± 8.3	.92	−7.6 ‐ 6.9	.02
Emotional eating clearly labelled	4.2 ± 2.4	84.2 ± 18.7	32.5 ± 6.7	2.4 ± 0.8	81.9 ± 21.1	32.8 ± 8.4	.53	−9.7 ‐ 4.9	.12
Emotional eating diffuse	3.6 ± 0.5	82.2 ± 18.4	32.0 ± 6.7	1.9 ± 0.7	82.5 ± 21.4	32.9 ± 8.5	.94	−6.7 ‐ 7.2	.01

Abbreviations: CI, confidence interval; DEBQ, Dutch Eating Behaviour Questionnaire; EWL, excess weight loss; TWL; total weight loss; SD, Standard Deviation.

^a^ High score emotional eating overall: males ≥2.6, females ≥3.3. High score emotional eating in response to clearly labelled emotions: males ≥2.5, females ≥3.1. High score emotional eating in response to diffuse emotions: males ≥2.7, females ≥3.7.

^b^ Low score emotional eating overall: males <2.6, females <3.3. High score emotional eating in response to clearly labelled emotions: males <2.5, females <3.1. High score emotional eating in response to diffuse emotions: males <2.7, females <3.7.

^c^ Based on independent samples t‐test between EWL in participants with high and low scores of emotional eating.

The regression model with overall emotional eating scores revealed that only initial BMI was a significant predictor for EWL (*β* = −0.36, 95% CI [−2.05, −0.84]) after adjusting for covariates (Table 3). Emotional eating in response to diffuse emotions showed, after adjusting for covariates, a negative association with EWL (*β* = −0.16, 95% CI [−1.57, −0.01]). The covariates accounted for 15.3%, 15.2% and 17.0% of the variance in EWL in the group of overall emotional eating, clearly labelled and diffuse emotions. Table 4 illustrates the regression model with overall emotional eating scores and TWL as the dependent variable. This analysis showed that only initial BMI was a significant predictor for TWL after adjusting for covariates (*β* = 0.30, 95% CI [0.24, 0.71]).

**TABLE 3:** Multiple linear regression analysis for predictors of EWL 2 years after surgery

		Emotional eating overall		Emotional eating in response to clearly labelled emotions		Emotional eating in responseto diffuse emotions	
		*Beta coefficient*	*P* value	*Beta coefficient*	*P* value	*Beta coefficient*	*P* value
Model 1^b^	Emotional eating	−.03	.71	−.02	.76	−.09	.24
Model 2^c^	Emotional eating	−.06	.49	−.05	.53	−.12	.15
	Anxiety	.00	1.00	.00	.99	.01	.90
	Depression	.12	.16	.11	.16	.12	.13
Model 3^d^	Emotional eating	−.06	.43	−.05	.51	−.16	.048^a^
	Anxiety	−.03	.66	−.04	.64	−.03	.74
	Depression	.07	.35	.07	.36	.08	.27
	Age	−.06	.44	−.06	.43	−.05	.52
	Gender	.06	.40	.06	.42	.07	.33
	Marital status	−.01	.92	−.01	.91	−.00	.96
	Diabetes mellitus type 2	−.08	.31	−.08	.32	−.11	.16
	Initial BMI	−.36	<.001^a^	−.35	<.001^a^	−.36	<.001^a^
	Preoperative psychological intervention	−.05	.50	−.05	.49	−.04	.62

*Note:* Dependent variable: %EWL 2‐year after surgery.

Abbreviation: BMI, body mass index.

^a^ *P* value  ≤ .05.

^b^ Model 1: predictor emotional eating (overall or in response to clearly labelled, or in response to diffuse emotions).

^c^ Model 2: predictor emotional eating (overall or in response to clearly labelled, or in response to diffuse emotions), anxiety, and depression.

^d^ Model 3: predictor emotional eating (overall or in response to clearly labelled, or in response to diffuse emotions), anxiety, depression, age, gender, marital status, diabetes mellitus type 2, BMI and preoperative psychological intervention.

**TABLE 4:** Multiple linear regression analysis for predictors of TWL 2 years after surgery

		Emotional eating overall		Emotional eating in response to clearly labelled emotions		Emotional eating in responseto diffuse emotions	
		*Beta coefficient*	*P* value	*Beta coefficient*	*P* value	*Beta coefficient*	*P* value
Model 1^b^	Emotional eating	−.04	.65	−.03	.66	−.10	.18
Model 2^c^	Emotional eating	−.04	.65	−.04	0.64	−.11	.18
	Anxiety	−.05	.56	−.05	0.55	−.04	.65
	Depression	.05	.58	.05	0.58	.05	.49
Model 3^d^	Emotional eating	−.04	.65	−.04	.65	−.12	.15
	Anxiety	−.06	.43	−.06	.42	−.05	.48
	Depression	.07	.37	.07	.37	.08	.30
	Age	−.09	.24	−.09	.24	−.08	.29
	Gender	.03	.75	.03	.74	.03	.65
	Marital status	.02	.76	.02	.76	.03	.72
	Diabetes mellitus type 2	−.10	.19	−.10	.19	−.13	.11
	Initial BMI	.30	<.001^a^	.30	<.001^a^	.29	<.001^a^
	Preoperative psychological intervention	−.05	.49	−.05	.49	−.04	.59

*Note:* Dependent variable: %TWL 2‐year after surgery.

Abbreviation: BMI, body mass index.

^a^ *P* value  ≤ .05.

^b^ Model 1: predictor emotional eating (overall or in response to clearly labelled, or in response to diffuse emotions);

^c^ Model 2: predictor emotional eating (overall or in response to clearly labelled, or in response to diffuse emotions), anxiety, and depression;

^d^ Model 3: predictor emotional eating (overall or in response to clearly labelled, or in response to diffuse emotions), anxiety, depression, age, gender, marital status, diabetes mellitus type 2, BMI and preoperative psychological intervention.

## 4 DISCUSSION

Earlier research shows that there has been little agreement on emotional eating as predictor of weight loss outcomes after bariatric surgery. The current study aimed to (1) investigate the association between emotional eating and EWL, and TWL; (2) explore the differential impact of emotional eating in response to clearly labelled and diffuse emotions on EWL and TWL because these are two distinguished types of emotional eating. With regard to the first aim of this study, our results showed no association between preoperative emotional eating and EWL/TWL 2 years after RYGB. In a separate analysis classifying high and low emotional eaters, there were no differences found between EWL and TWL. Regarding the second aim of this study our regression analysis showed that, when correcting for multiple covariates, emotional eating in response to diffuse emotions had a negative impact on EWL, although this was not applicable for TWL. It should be noted that the finding was borderline significant (p 0.048) and in presence of a confounder (initial BMI).

There are two remarkable findings when reviewing characteristics of the study population. First of all, there were only 11 participants defined as non‐responders limiting further analysis of EWL in responders and non‐responders. The low rate of non‐response contradicts the rate of 25% to 35% reported in literature.^1^, ^2^, ^8^ This finding could possibly be explained by the large set of excluded participants (43%) as these participants may have experienced more non‐response. Non‐response may have reduced motivation to attend follow‐up appointments which could have contributed to missing data. Secondly, a large amount of participants (36%) received a psychological intervention preoperatively. This intervention may have altered levels of emotional eating postoperatively and consequently effected weight loss outcomes. However, participation in this intervention was not associated with EWL or TWL based on the regression analysis performed.

When considering all demographic and psychological variables tested in the regression analysis, initial BMI showed a negative association with EWL which is in line with other literature.^19^, ^20^ Initial BMI showed a positive association with TWL which is also supported by literature.^21^ This difference can be explained by the fact that EWL is influenced by baseline BMI because it relies on an ideal body weight (ie, BMI 25 kg/m^2^), whereas TWL is less influenced by BMI.^20^ There was no association between anxiety and EWL/TWL, nor between depression and EWL/TWL. These findings are not consistent across studies as some studies did find associations, yet the opposite has also been found.^22^, ^23^, ^24^, ^25^, ^26^

Not finding an association between depression and EWL/TWL, and between anxiety and EWL/TWL could be explained by the use of the SCL‐90. This is a self‐report questionnaire that measured psychological symptoms and distress over the past week and was not designed to classify psychological or psychiatric disorders. Another explanation might be that the level of psychopathology in the cohort was not representative for the level of psychopathology in the population of individuals with obesity. Namely, bariatric candidates with high levels of preoperative psychopathology are more likely to be denied for surgery.

This study presents limitations that can be partly traced back to using the DEBQ emotional eating scale. Due to the DEBQ being a self‐report questionnaire, response bias may have occurred. Participants may have underreported the level of emotional eating in order to be eligible for bariatric surgery. Alternatively, participants may have lacked insights into their own eating behaviour or emotions which may have influenced their questionnaire response. It is also important to note that the DEBQ assesses desire to eat in response to emotions as opposed to actual eating in response to emotions. It is possible that not assessing actual eating may have biased the results. Besides these limitations, it should be mentioned that this study lacked examination of emotional eating postoperatively and it therefore unknown how emotional eating developed over time. Moreover, the follow‐up time of 2 years may not have been long enough to detect non‐response. Lastly, this study suffered from a poor response rate as 43% of the participants were excluded from the final analysis.

In order to develop a full picture of the relationship between emotional eating and postoperative weight loss, additional studies will be needed. Prospective studies in large cohorts (eg, participants undergoing RYGB, as well as sleeve gastrectomy) should examine the effect of preoperative emotional eating on long‐term weight loss outcomes including non‐response. Longitudinal studies could gain insights in the development of emotional eating over time and how this may contribute to non‐response. The yields of additional studies may result in a number of practical implications like improvement of preoperative evaluation and subsequent patient selection.

In conclusion, the current study found no association between preoperative emotional eating and EWL, nor between preoperative emotional eating and TWL 2 years after RYGB. When focusing on emotional eating specifically in response to diffuse emotions, it seemed that emotional eating had a negative impact on EWL although this was not applicable for TWL. This study faced multiple limitations such as response bias, underreporting bias and a poor response rate thereby hampering clinical guidance.

## CONFLICT OF INTEREST

The authors declare that they have no conflict of interest.

## AUTHORS CONTRIBUTION

Authors J. Schellekens, D.S. Bonouvrie, M. van de Wal designed the study and wrote the study protocol. L. Janssen, F.M.H. van Dielen, W.K.G. Leclercq and AL participated in conducting the study. J. Schellekens and M.M. Romeijn conducted the statistical analysis. J. Schellekens wrote the first draft of the manuscript, M.M. Romeijn revised the manuscript multiple times. All authors have contributed to, and have approved the final manuscript.

## Supporting information

**Table S1** Gender specific cut‐off scores DEBQ for high vs low emotional eating.
