# Supplementary material for: Emotional eating as predictor of weight loss 2 years after Roux‐en‐Y gastric bypass
Source: Clin Obes. 2021 May 30;11(4):e12458. doi: 10.1111/cob.12458 (PMC8365652; doi:10.1111/cob.12458)
Supplement: Supplementary file 1 — Table S1 Gender specific cut‐off scores DEBQ for high vs low emotional eating. [file COB-11-e12458-s001.docx]

Supplementary Table 1. Gender specific cut-off scores DEBQ for high versus low emotional eating.

| 1a. ≥2.6 for males and ≥3.3 for females was classified as a high score for emotional eating  1b. <2.6 for males and <3.3 for females was classified as a low score for emotional eating  2a. ≥2.5 for males and ≥3.1 for females was classified as a high score for emotional eating in response to clearly labelled emotions  2b. <2.5 for males and <3.1 for females was classified as a low score for emotional eating in response to clearly labelled emotions  3a. ≥2.7 for males and ≥3.7 for females was classified as a high score for emotional eating in response to diffuse emotions  3b. <2.7 for males and <3.7 for females was classified as a high score for emotional eating in response to diffuse emotions. |
| --- |
